# Natural Terpenoids from *Ambrosia* Species Are Active *In Vitro* and *In Vivo* against Human Pathogenic Trypanosomatids

**DOI:** 10.1371/journal.pntd.0002494

**Published:** 2013-10-10

**Authors:** Valeria P. Sülsen, Silvia I. Cazorla, Fernanda M. Frank, Laura C. Laurella, Liliana V. Muschietti, Cesar A. Catalán, Virginia S. Martino, Emilio L. Malchiodi

**Affiliations:** 1 Cátedra de Farmacognosia, IQUIMEFA (UBA-CONICET), Facultad de Farmacia y Bioquímica, Universidad de Buenos Aires, Buenos Aires, Argentina; 2 Cátedra de Inmunología, IDEHU (UBA-CONICET), Facultad de Farmacia y Bioquímica, Buenos Aires, Argentina and Instituto de Microbiología y Parasitología Médica, IMPaM (UBA-CONICET), Facultad de Medicina, Buenos Aires, Argentina; 3 INQUINOA-CONICET, Instituto de Química Orgánica, Facultad de Bioquímica, Química y Farmacia, Universidad Nacional de Tucumán, San Miguel de Tucumán, Argentina; Northeastern University, United States of America

## Abstract

Among the natural compounds, terpenoids play an important role in the drug discovery process for tropical diseases. The aim of the present work was to isolate antiprotozoal compounds from *Ambrosia elatior* and *A. scabra*. The sesquiterpene lactone (STL) cumanin was isolated from *A. elatior* whereas two other STLs, psilostachyin and cordilin, and one sterol glycoside, daucosterol, were isolated from *A. scabra*. Cumanin and cordilin were active against *Trypanosoma cruzi* epimastigotes showing 50% inhibition concentrations (IC_50_) values of 12 µM and 26 µM, respectively. Moreover, these compounds are active against bloodstrean trypomastigotes, regardless of the *T. cruzi* strain tested. Psilostachyin and cumanin were also active against amastigote forms with IC_50_ values of 21 µM and 8 µM, respectively. By contrast, daucosterol showed moderate activity on epimastigotes and trypomastigotes and was inactive against amastigote forms. We also found that cumanin and psilostachyin exhibited an additive effect in their trypanocidal activity when these two drugs were tested together. Cumanin has leishmanicidal activity with growth inhibition values greater than 80% at a concentration of 5 µg/ml (19 µM), against both *L. braziliensis* and *L. amazonensis* promastigotes. In an *in vivo* model of *T. cruzi* infection, cumanin was more active than benznidazole, producing an 8-fold reduction in parasitemia levels during the acute phase of the infection compared with the control group, and more importantly, a reduction in mortality with 66% of the animals surviving, in comparison with 100% mortality in the control group. Cumanin also showed nontoxic effects at the doses assayed *in vivo*, as determined using markers of hepatic damage.

## Author Summary

In addition to the primary metabolism necessary for life, plants have a secondary metabolism that generates compounds, which aid in their growth and development. A common role of secondary metabolites is defense mechanisms to fight off animals, pest and pathogens. Pharmacognosy takes advantage of the rich source of compounds produced by plants, selecting and processing natural products for medicinal use, resulting in a wide range of anticancer, anti-inflammatory and anti-infective drugs currently in use. Chagas disease and leishmaniasis are parasitic diseases caused by protozoa transmitted by sucking insects. According to the World Health Organization (WHO) they are considered as Neglected Tropical Diseases that are especially endemic in low-income populations in developing countries. The drugs currently in use were developed more than 50 years ago and have severe drawbacks. The authors found that chemical compounds called sesquiterpene lactones, obtained from plants of the Asteraceae family, have anti-protozoan activity against different parasite stages. The identified compounds were also able to control parasitic infection in mice without any toxic effects. Thus, these sesquiterpenes lactones could be interesting chemical compounds having pharmacological properties, whose chemical structure could be modified leading to potent and safer drugs to treat these parasitoses.

## Introduction

Natural products have been a major source of drugs mainly for treating infectious diseases and cancer [1]. About 75% of anti-infective drugs approved from 1981 to 2002 are derived from natural sources. Many of them were isolated from plants and have shown antiparasitic activity. The first antimalarial drug, quinine, was isolated from *Cinchona* spp. and led to the development of other antimalarial drugs such as chloroquine, which is currently in use. More recently, the sesquiterpene lactone (STL) artemisinin has been isolated from the Chinese plant *Artemisia annua*, which has been used for over 2000 years to treat malaria. At present, this natural compound and its derivatives are used for treating chloroquine-resistant malaria.

Both Chagas disease and leishmaniasis are protozoan diseases that cause significant morbidity and mortality in Latin America, whereas leishmaniasis also worldwide. According to the World Health Organization (WHO), they are considered, among others, Neglected Tropical Diseases (NTDs) mainly affecting poor people in developing countries [2]. These parasitoses are often forgotten by governments and the pharmaceutical industry, due to economic reasons and a relatively limited market. Therefore, the development of new drugs for the treatment of these parasitic diseases remains a highly desirable goal.

Chagas' disease or American trypanosomiasis is caused by the protozoan parasite *Trypanosoma cruzi*, which is transmitted by blood-sucking insects. This parasitic disease affects 8 million people, mostly in endemic areas of Latin America, but has now spread to other continents [3]. Up to 30% of patients develop heart failure and people usually die from sudden death caused by arrhythmias. The chronic phase of the disease can cause damage to the esophagus, colon or the autonomic nervous system in more than 10% of patients. It is estimated that Chagas' disease killed more than 10,000 people in 2008 [3]. In Argentina, 1.5 to 2 million people are affected and it is estimated that 15 people die of this parasitosis every week [4].

Leishmaniasis is caused by kinetoplastids from the genus *Leishmania* and is transmitted by sand flies. WHO estimates that almost 12 million people worldwide are infected with *Leishmania* spp. and 350 million are at risk of contracting this parasitic disease [5]. Cutaneous leishmaniasis is endemic in Northern Argentina covering an area of 500,000 km^2^ where it is common to find people coinfected with *T. cruzi* and *Leishmania* sp. [6]. Its incidence has increased during the last two decades mainly due to *Leishmania braziliensis* [7]. The clinical manifestations of these parasitoses depend on the *Leishmania* species involved, presenting three different clinical forms: cutaneous, mucocutaneous and visceral leishmaniasis.

Most of the drugs currently in use for the treatment of American trypanosomiasis and leishmaniasis have severe drawbacks. The available treatments for Chagas disease are limited to the nitroaromatic compounds, benznidazole and nifurtimox, which were released in the 1970s. Even though these two drugs are active in the acute stage of infection, they are ineffective in the treatment of the chronic phase. They have toxic side effects and are not active against all *T. cruzi* strains [8]. The current antileishmanial therapy includes the use of pentavalent antimonials, amphotericin B, miltefosine or paromomycin, which have disadvantages in terms of the route of administration, parasite resistance, cost, teratogenic effects, length of treatment and toxicity [9]. Among the natural compounds, terpenoids display a wide range of biological activities such as anticancer and anti-inflammatory actions and are effective against infective agents such as viruses, bacteria and parasites [10], [11]. Several terpenoids have been reported to have trypanocidal and leishmanicidal activities [12]–[15]. Recent research has shown the potential role of terpenoids as promising compounds against neglected protozoan diseases such as Chagas' disease and leishmaniasis [16], [17]. An exhaustive and updated revision has been recently performed by Schmidt and coworkers [18]. These authors revised the antiprotozoal activities of the major biogenetic subclassses of terpenes focusing on STLs, diterpenes and triterpenes. Within the STLs, germacranolides, guaianolides, xanthanolides and pseudoguaianolides, have exhibited significant antiprotozoal activity, showing the potential of this class of compounds [18].

In the last decades, Asteraceae has been regarded as a promising family of plants because of the amount and variety of active compounds produced by the secondary metabolism. STLs and triterpenes isolated from this family have been reported as having trypanocidal and leishmanicidal activities [16]–[18], [19]. In Argentina, the genus *Ambrosia* that belongs to this family is represented by three species: *A. tenuifolia*, *A. scabra* and *A. elatior*. Considering the limitations of current therapies for Chagas disease and leishmaniasis and our previous promising findings on the antiprotozoal activity of terpenoids from the genus *Ambrosia* [20]–[23], the aim of the present work was to isolate further bioactive compounds from *A. elatior* and *A. scabra*.

## Materials and Methods

### Plant material

The aerial parts of *Ambrosia elatior* L. (BAF 707) and *Ambrosia scabra* Hook. & Arn. (Asteraceae) (BAF 711) were collected in Buenos Aires Province, Argentina in May 2009. The botanical identification was performed by Dr. Gustavo Giberti and a voucher specimen of each species was deposited at the Museo de Farmacobotánica, Facultad de Farmacia y Bioquímica, Universidad de Buenos Aires.

### Parasites

*Trypanosoma cruzi* epimastigotes (RA and K98 strains) were grown in a biphasic medium. Cultures were routinely maintained by weekly passages at 28°C. *T. cruzi* bloodstream trypomastigotes from RA and K98 strains [24] were obtained from infected CF1 mice by cardiac puncture at the peak of parasitemia on day 15 postinfection. Trypomastigotes were routinely maintained by infecting 21-day-old CF1 mice. *T. cruzi* amastigotes were obtained from cultured cells infected with tripomastigotes. *Leishmania braziliensis* and *Leishmania amazonensis* promastigotes (MHOM/BR/75/M2903 and MHOM/BR/75/M2269 strains, respectively) were grown in liver infusion tryptose medium (LIT). Cultures were routinely maintained by weekly passages at 26°C. Parasites were passaged 24 or 48 h previous to the experiments.

### Animal ethics statement

Inbred male C3H/HeN mice were nursed at the Microbiology Department, Faculty of Medicine, University of Buenos Aires. All procedures requiring animals were performed in agreement with institutional guidelines and were approved by the Review Board of Ethics of IDEHU, CONICET, and conducted in accordance with the Guide for the Care and Use of Laboratory Animals of the National Research Council of Argentina [25].

### Plant extracts

Extraction of the aerial parts of *A. elatior* and *A. scabra* was done by maceration with dichloromethane∶methanol (1∶1), as previously described [22].

### Fractionation of *Ambrosia elatior* extract

The organic extract of *A. elatior* (AE-OE) was fractionated by column chromatography on Silica gel 60 with a gradient of hexane, ethyl acetate and methanol. Nine fractions (F_1AE_–F_9AE_) of 500 ml each were collected and taken to dryness. Each fraction was tested for trypanocidal activity on *T. cruzi* epimastigotes. Fractions F_5AE_, F_6AE_ and F_7AE_ were taken with ethyl acetate and afforded a crystalline compound named compound A, which was assayed for trypanocidal and leishmanicidal activities.

### Isolation of compounds from fraction F_5AS_ of *Ambrosia scabra*

Fractionation of organic extract of *A. scabra* has been previously described [23]. A pure compound, compound B, was obtained from fractions F_5AS(70–74)_ by crystallization from ethyl acetate. Compound C was obtained as a white crystalline precipitate from fractions F_5AS(75–77)_, while fractions F_5AS(125–135)_ afforded a white amorphous powder (compound D).

### Compound identification

The structure elucidation of compounds A–D was performed by proton nuclear magnetic resonance (^1^H NMR) and carbon NMR (^13^C NMR) (Inova NMR spectrometer; Varian, Palo Alto, CA) 500 MHz in CDCl_3_ (for compounds A, B and C) and CDCl_3_:CD_3_OD (8∶2) (for compound D), heteronuclear single quantum correlation (HSQC); heteronuclear multiple bond correlation (HMBC); correlated spectroscopy (COSY); electron impact-mass spectrometry (EI-MS) (Agilent 5973) and infrared spectroscopy (Bruker FT-IR IFS25).

### *In vitro* trypanocidal and leishmanicidal activity assay

Growth inhibition of *T. cruzi* epimastigotes and *Leishmania* spp. promastigotes was evaluated by a [^3^H] thymidine uptake assay as previously described [20]. Parasites were adjusted to a cell density of 1.5×10^6^/ml and cultured in the presence of *A. elatior* organic extract, the fractions and purified compounds for 72 h at final concentrations ranging from 1 to 100 µg/ml. Benznidazole (5 to 20 µM; Roche) and Amphotericin B (0.27–1.6 µM; ICN) were used as positive controls (data not shown). The percentage of inhibition was calculated as 100−{[(cpm of treated parasites)/(cpm of untreated parasites)]×100}.

The trypanocidal effect of the pure compounds was also tested on bloodstream trypomastigotes of RA and K98 strains as previously described [20]. Briefly, mouse blood containing trypomastigotes was diluted in complete liver infusion tryptose medium to adjust the parasite concentration to 1.5×10^6^/ml. Parasites were seeded (150 µl/well) by duplicate into a 96-well microplate, and 2 µl of each compound/well at different concentrations or control drug (benznidazole) was added per well. Plates were incubated for 24 h and the remaining live parasites were counted on a hemocytometer. Results are expressed as [live parasites in wells after compound treatment/live parasites in untreated wells]×100.

To evaluate the effect of the compounds on intracellular forms of *T. cruzi*, 96-well plates were seeded with nonphagocytic Vero cells at 5×10^3^ per well in 100 µL of culture medium and were incubated for 24 h at 37°C in a 5% CO_2_ atmosphere. Cells were washed and infected with transfected blood trypomastigotes expressing β-galactosidase [26] at a parasite/cell ratio of 10∶1. After 24 h of coculture, plates were washed twice with PBS to remove unbound parasites and each pure compound was added at different concentrations in 150 µl of fresh complete RPMI medium without phenol red (Gibco, Rockville, MD). Controls included infected untreated cells (100% infection control) and uninfected cells (0% infection control). The assay was developed by the addition of chlorophenolred-β-D-galactopyranoside (CPRG) (100 µM) and 1% Nonidet P40, 5 days later. Plates were then incubated for 4–6 h at 37°C and the absorbance was measured at 595 nm in a microplate reader (Bio-Rad Laboratories, Hercules, CA). Percentage inhibition was calculated as 100–{[(absorbance of treated infected cells)/(absorbance of untreated infected cells)×100} and the IC_50_value was estimated.

### Drug interaction

Compounds A and B were combined with each other, as well as compound A and benznidazole, to evaluate a potential interaction among them. Trypanocidal activity was evaluated on RA epimastigotes by a [^3^H] thymidine uptake assay as previously described [20]. The fractional inhibitory concentrations (FICs) were calculated as the ratio of the IC_50_ of one compound in combination and the IC_50_ of the compound alone. The FIC index (FICI) for the two compounds was the FIC of compound A plus the FIC of compound B. The fractional inhibitory concentration index (FICI) was interpreted as follows: FICI≤0.5 synergy, FICI>4.0 antagonism, FICI = 0.5–4 addition [27].

### *In vitro* cytotoxicity assay

Vero cells were assayed to determine viability by the MTT method [22]. Cells (5×10^5^) were settled at a final volume of 150 µl in a flat-bottom 96-well microplate and were cultured at 37°C in a 5% CO_2_ atmosphere in the absence or presence of increasing concentrations of the pure compounds. After 24 h, 3-(4,5-dimethylthiazol-2yl)-2,5-diphenyltetrazolium bromide (MTT) was added at a final concentration of 1.5 mg/ml. Plates were incubated for 2 h at 37°C. The purple formazan crystals were completely dissolved by adding 150 µl of ethanol and the absorbance was detected at 595 nm in a microplate reader. Results were calculated as the ratio between the optical density in the presence and absence of the compound multiplied by 100.

### *In vivo* trypanocidal activity of compound A

Groups of five C3H/HeN male mice (6 to 8 weeks old; 27.2±0.9 g) were infected with 500 bloodstream *T. cruzi* trypomastigotes by the intraperitoneal route. Five days after infection, the presence of circulating parasites was confirmed by the microhematocrit method [23]. Mice were treated daily with compound A or benznidazole (1 mg/kg of body weight/day) for five consecutive days (days 5 to 10 postinfection) by the intraperitoneal route. Drugs were resuspended in 0.1 M phosphate buffered saline (PBS, pH 7.2), and this vehicle was also employed as a negative control. Levels of parasitemia were monitored every 2 days in 5 µl of blood diluted 1∶5 in lysis buffer (0.75% NH_4_Cl, 0.2% Tris, pH 7.2) by counting parasites in a Neubauer chamber. The number of deaths was recorded daily.

### *In vivo* toxicity

Groups of five C3H/HeN male uninfected mice were treated with PBS or compound A as described above, in order to evaluate the potential *in vivo* toxicity of the compound. On day 7 post treatment, blood samples were collected by cardiac puncture. Serum activities of alanine aminotransferase (ALT), aspartate aminotransferase (AST) and lactate dehydrogenase (LDH) were determined as markers of hepatic damage. Assays were carried out by ultraviolet spectrophotometry following the specifications of the kit's manufacturer (Wiener Lab, Buenos Aires, Argentina).

### Statistical analysis

The results are presented as mean±SEM. The level of statistical significance was determined by using one-way analysis of variance (ANOVA), with GraphPad Prism 5.0 software (GraphPad Software Inc., San Diego, CA). Long rank test was used for survival curves. Comparisons were referred to the control group. P values of <0.05 were considered significant.

## Results

### Fractionation of *Ambrosia elatior* organic extract

The dichloromethane∶methanol organic extract (AE-OE) from the aerial parts of *Ambrosia elatior* was evaluated *in vitro* against *T. cruzi* epimastigotes. This extract was active with a growth inhibition of 93.7±2.0% at a concentration of 100 µg/ml. The extract fractionation by chromatographic techniques using Silica gel 60 with a gradient of hexane, ethyl acetate and metanol yielded nine fractions (F_1AE_ to F_9AE_), which were assayed for their *in vitro* trypanocidal activity on epimastigotes at 100, 10 and 1 µg/ml (**Table 1**). The results of this experiment showed that at a concentration of 10 µg/ml, fractions F_5AE_, F_6AE_ and F_7AE_ were the most active with percentages of growth inhibition of 94.7±0.5%, 75.3±4.8% and 76.8±1.4%, respectively (**Table 1**).

**Table 1:** *In vitro* trypanocidal activity of organic extract and fractions of *Ambrosia elatior* on RA epimastigotes of *T. cruzi*.

Extract/fractions	% Growth Inhibition		
	100 µg/ml	10 µg/ml	1 µg/ml
AE-OE	93.7±2.0	35.1±0.5	8.8±3.1
F_1AE_	1.4±6.0	2.8±0.9	3.1±0.2
F_2AE_	93.6±0.7	34.5±1.8	33.5±3.0
F_3AE_	93.7±0.9	49.6±6.9	9.2±7.9
F_4AE_	96.2±0.3	37.5±1.5	8.3±9.5
F_5AE_	96.5±0.2	94.7±0.5	41.0±2.9
F_6AE_	91.8±3.1	75.3±4.8	31.4±5.5
F_7AE_	86.3±2.6	76.8±1.4	31.1±14.1
F_8AE_	79.1±2.0	32.0±4.2	29.7±11.0
F_9AE_	40.3±5.2	25.2±3.0	20.7±3.7

Results are expressed as mean ± S.E.M. (n = 3).

**Figure 1:**
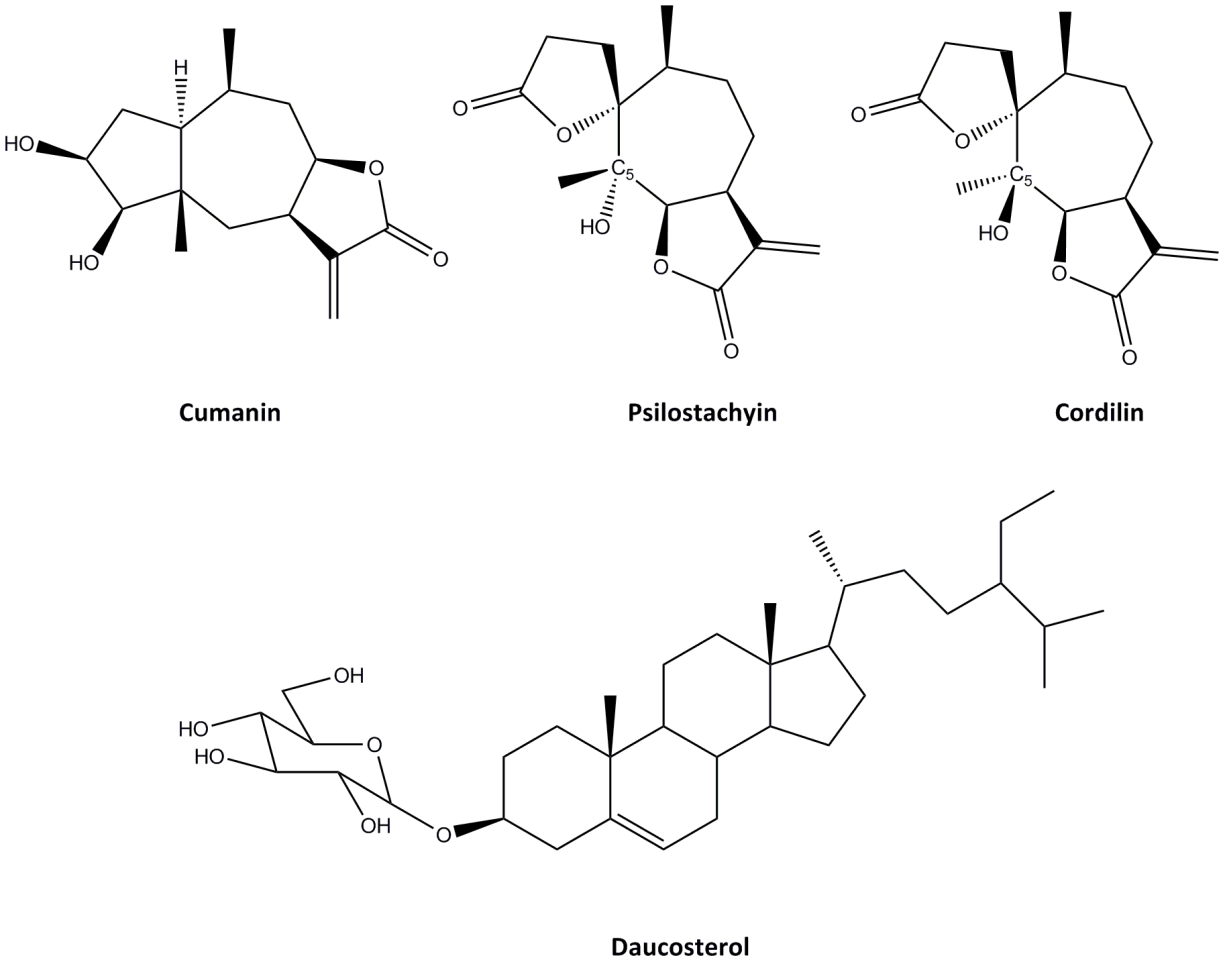
Chemical structures of the sesquiterpene lactones cumanin, psilostachyin and cordilin and the sterol glycoside daucosterol.

A pure compound (compound A) was further isolated from fractions F_5AE_, F_6AE_ and F_7AE_. It was identified by spectroscopic methods as the STL 1α,3β,4β,5β,8β,10β-3,4-dihydroxy-11(13)-pseudoguaien-12,8-olide or **cumanin** (**Figure 1**).

### Isolation of compounds from fraction F_5AS_ of *Ambrosia scabra*

An exhaustive search in the active fraction F_5AS_ from *A. scabra* resulted in the isolation of three other compounds, B, C and D. The analyses of spectroscopic data allowed to identify compounds B and C as the STLs (2′R,3aS,6s,8s,8aR)-octahydro-8-hydroxy-6,8-dimethyl-3-methylene-spiro[7H-cyclohepta[b]furan-7,2′(5′H)-furan]-2,5′(3H)-dione **psilostachyin** and its 5-epimer **cordilin** or epipsilostachyin, respectively. Compound D was identified as the sterol glycoside **sitosterol**-**3-O-β-D-glucopyranoside** or **daucosterol** (**Figure 1**).

### Inhibition of *T. cruzi* epimastigotes

**Figure 2:**
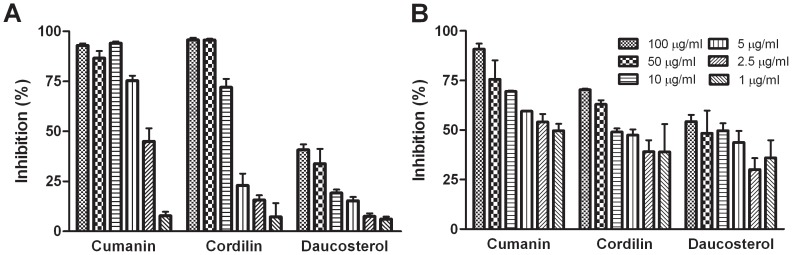
Inhibition of *T. cruzi* epimastigotes by cumanin, cordilin and daucosterol. Growth inhibition of parasites was evaluated by a [3H] thymidine uptake assay. Epimastigotes of (**A**) RA and (**B**) K98 strain, were adjust at 1.5×10^6^/ml and cultured for 72 h at 28°C in the presence of the compounds at a final concentration ranging from 100 - 1 µg/ml. The percentage of inhibition was calculated as 100−[(cpm of treated parasites)/(cpm of untreated parasites)]×100. Values represent mean ± SEM from three independent experiments carried out in triplicate.

We analyzed the antiparasitic activity of the isolated compounds at different stages of *Trypanosoma cruzi* and *Leishmania* sp. The results of the trypanocidal activity of cumanin, cordilin and daucosterol on *T. cruzi* epimastigote forms are shown in **Figure 2**. The STLs were active with 50% inhibition concentration (IC_50_) values of 12 µM and 4 µM for cumanin, and 26 µM and 44 µM for cordilin against RA and K98 epimastigotes, respectively. Daucosterol exhibited lower activity with a growth inhibition of 40.7±2.8% at 100 µg/ml (174 µM).

### Drug interaction by the isobologram analysis

**Figure 3:**
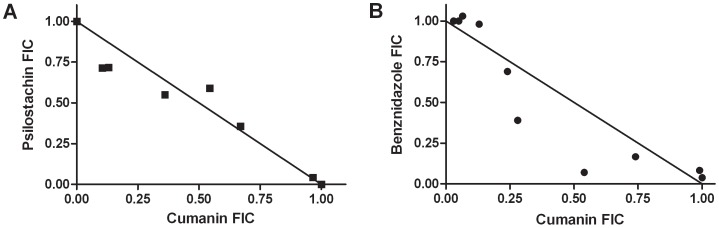
Isobologram describing the effect of the combination of (A) cumanin and psilostachyin and, (B) cumanine and benznidazole, on epimastigotes of *T. cruzi*. Growth inhibition of epimastigotes was evaluated by a [3H] thymidine uptake assay. RA epimastigotes were cultured for 72 h in the presence of different combinations of both compounds ranging from 0–5 µg/ml. The fractional inhibitory concentrations of drugs (FIC) were calculated for each point, and an isobologram was plotted. The fractional inhibitory concentration index (FICI) was interpreted as follows: FICI≤0.5 synergy, FICI>4.0 antagonism, FICI = 0.5–4 addition.

The combinatory effect of the two most active STLs, psilostachyin and cumanin, on epimastigotes of *T. cruzi* was analyzed by an isobologram. The fractional inhibitory concentration index (FICI) was 0.97±0.11 (Mean±SD) for the combination of these drugs, indicating there is neither antagonism nor synergism; however, an additive effect could be assessed in the trypanocidal activity between cumanin and psilostachyin (**Figure 3**). We neither found antagonism nor synergism interaction between cumanin and benznidazole, the current reference drug to treat Chagas disease (FICI: 0.95±0.05) (Mean±SD) (**Figure 3**).

### Inhibition of *T. cruzi* trypomastigotes

**Figure 4:**
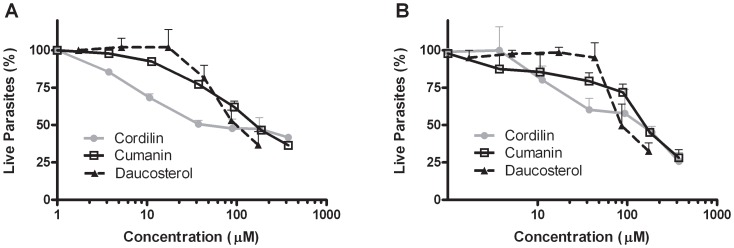
Effect of cumanin, cordilin and daucosterol against *T. cruzi* trypomastigotes. Bloodstream trypomastigotes diluted at 1.5×10^6^/ml in complete liver infusion tryptose medium, were seeded by duplicate in the presence of different concentration of each compound (0–376 µM, 0–375 µM and 0–174 µM, for cumanin, cordilin and daucosterol, respectively) and incubated at 4°C for 24 h. The remaining live parasites of (**A**) RA strain, and (**B**) K98 strain, in each sample was determined in 5 µl of cell suspension diluted 1/5 in lysis buffer (0.75% NH4Cl, 0.2% Tris, pH 7.2) and counted in a Neubauer chamber. Values represent mean ± SEM from three independent experiments carried out in duplicate.

The trypanocidal effect of the pure compounds was tested on bloodstream trypomastigotes obtained from infected mice. Parasites were seeded into a 96-well microplate and different concentrations of each compound were added to each well. After 24 h incubation, the remaining live parasites were counted on a hemocytometer. In **Figure 4** it can be appreciated that the two STLs, cumanin and cordilin, showed activity against RA trypomastigotes with IC_50_ values of 180 µM and 90 µM, respectively. The sterol glycoside was active with an IC_50_ of 142 µM.

In order to analyze the relevance of the compounds in different *T. cruzi* populations, K98 trypomastigotes were incubated in the presence of cumanin, cordilin and daucosterol, showing IC_50_ values of 170 µM, 83 µM and 184 µM, respectively (**Figure 4**).

### Inhibition of *T. cruzi* amastigotes

**Figure 5:**
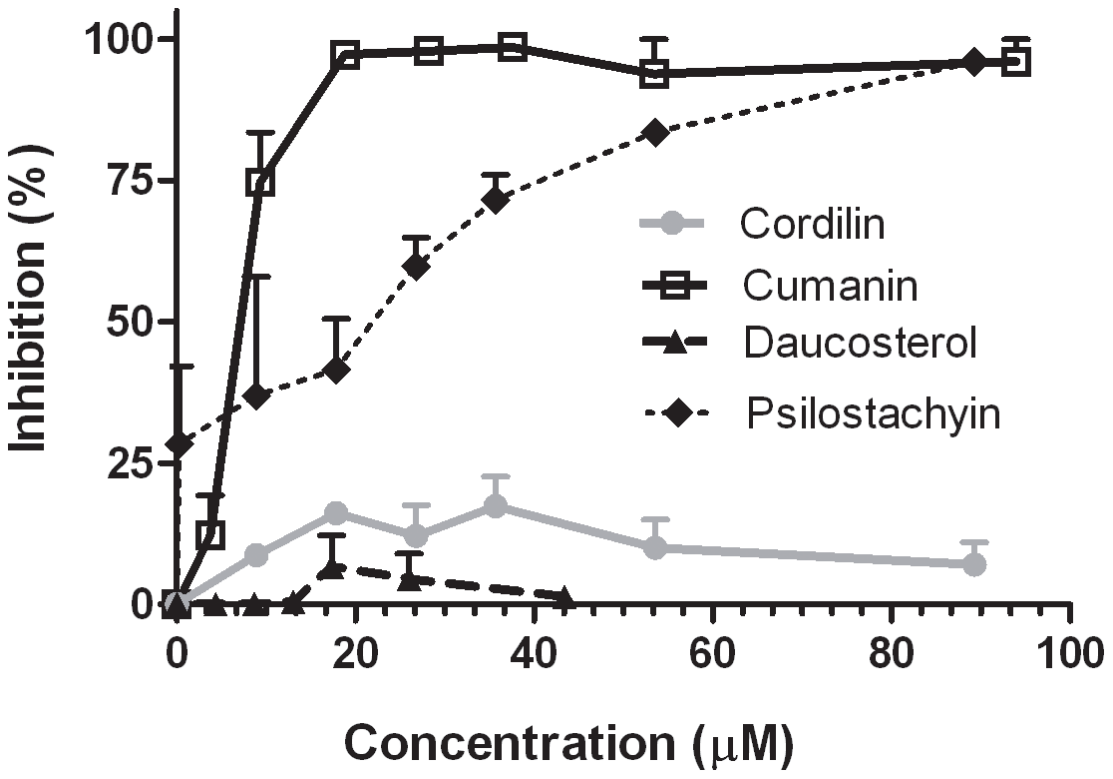
Inhibition of *T. cruzi* amastigotes by cumanin, psilostachyin, cordilin and daucosterol. Vero cells (5×10^3^ p/well) were seeded in a 96 well plates and infected 24 h later with transfected trypomastigotes expressing beta-galactosidase at a parasite cell ratio 10∶1. After 24 h of coculture, plates were washed and drug compounds were added at 0–94 µM, 0–96 µM, 0–96 µM and 0–43 µM, for cumanin, psilostachyin, cordilin, and daucosterol, respectively, in 150 µl of RPMI medium without phenol red. On day 6 p.i., the assays were developed by addition of CPRG (100 mM) and Nonidet P-40 (1%). Plates were incubated for 4–6 h at 37°C and quantitated at 570 nm. Controls included infected untreated cells (100% infection control). The percentage of inhibition was calculated as 100−{[(Absorbance of treated infected cells)/(Absorbance of untreated infected cells)]×100}.

To analyze the effect of the compounds on the replicative forms, Vero cells were infected with transfected trypomastigotes expressing the β-galactosidase gene. After 24 h incubation, all the parasites outside the cells were removed by washing and different concentrations of the compounds were added to the wells. Five days later the cells were disrupted with detergent and β-galactosidase activity was determined with chlorophenol red-β-D-galactopyranoside, as a direct estimation of the number of parasites. Since the effect of psilostachyin was not previously reported, the four isolated compounds were included in the assay. **Figure 5** shows that cumanin and psilostachyin were able to inhibit amastigote replication. Both STLs were active with approximately 95% inhibition at 25 µg/ml, and IC_50_s of 8 µM and 21 µM, respectively. Neither cordilin nor daucosterol were active against the intracellular forms of *T. cruzi*.

### Inhibition of *L. braziliensis and L. amazonensis* promastigotes

**Figure 6:**
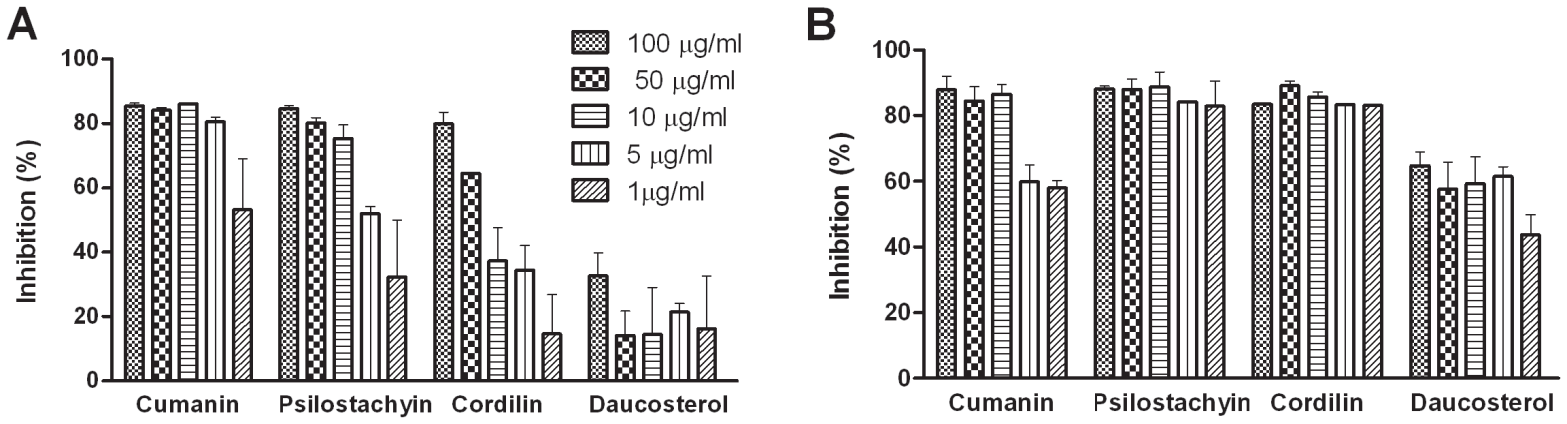
Inhibition of *Leishmania* promastigotes grown by cordilin, psilostachyin, cumanin and daucosterol. Promastigotes of (**A**) *L. amazonensis* and (**B**) *L. braziliensis*, were adjust at 1.5×10^6^/ml and cultured for 72 h at 26°C in the presence of 1–100 µg/ml of the compounds. [3H] thymidine was added for the last 16 h of culture. The percentage of inhibition was calculated as 100−[(cpm of treated parasites)/(cpm of untreated parasites)]×100. Result is representative of two independent experiments carried out in triplicate.

After the observed effect of cumanin, psilostachyin, cordilin and daucosterol on *T. cruzi*, we analyzed the inhibitory activity of these compounds on *Leishmania* promastigotes. The IC50 values against *L. amazonensis* were 3 µM, 10 µM and 55 µM, for cumanin, psilostachyin and cordilin, respectively. We found that cumanin revealed leishmanicidal activity with growth inhibition values greater than 80% at a concentration of 5 µg/ml (19 µM), against both *L. braziliensis* and *L. amazonensis* (**Figure 6**). Psilostachyin and cordilin displayed high leishmanicidal activity against *L. braziliensis* (82.8±7.8 and 83.03±0.01% at a concentration of 1 µg/ml, respectively).

These results showed that cumanin was active against the two species reported as the main cause of tegumentary leishmaniosis in Argentina: *L. braziliensis* and *L.amazonensis* [6].

### *In vivo* trypanocidal activity of cumanin

**Figure 7:**
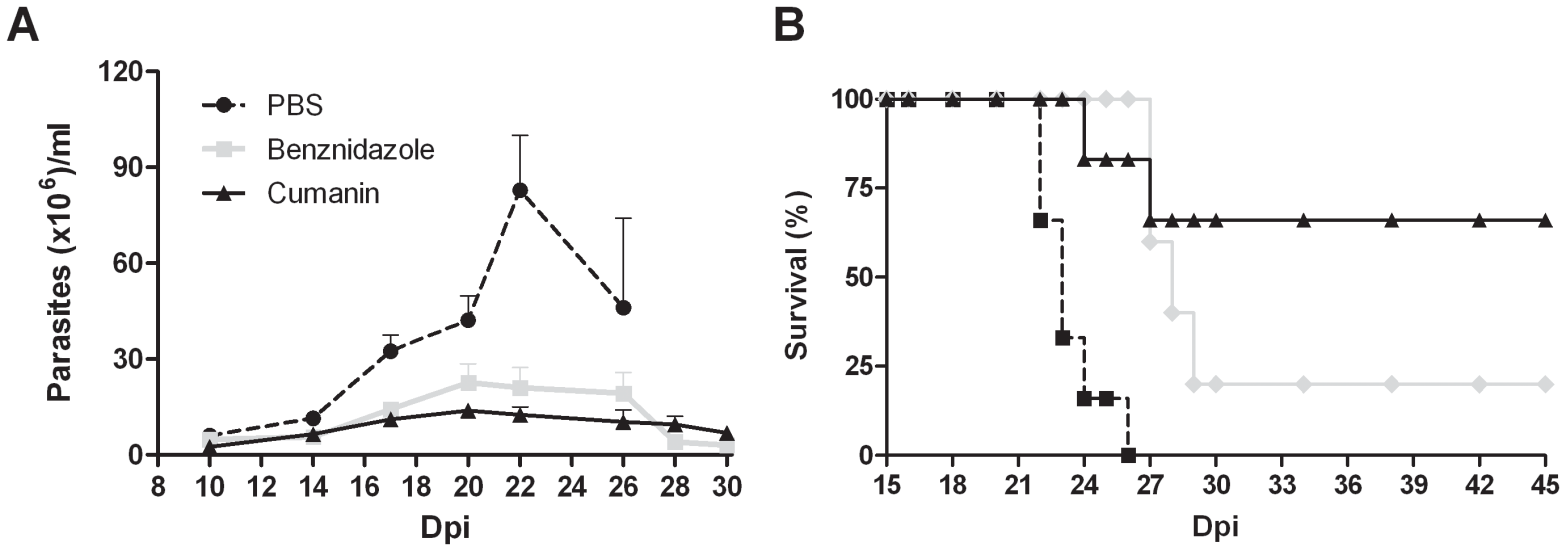
*In vivo* trypanocidal activity of cumanin: parasitemia levels (A) and survival curve (B) during the acute infection period. C3H/HeN female mice infected with 500 trypomastigotes were treated daily, by intraperitoneal route, with PBS, cumanin or benznidazole (1 mg/kg of body weight/day), starting on day 5 postinfection. Levels of parasitemia were monitored every 2 days in 5 µl of fresh blood from the tail vein, by counting parasites in a Neubauer chamber. The number of dead mice was recorded daily. Results are representative of three independent experiments.

To analyze the effect of the active compounds *in vivo*, a group of mice was inoculated with a deadly number of trypomastigotes of the RA strain and parasitemia was determined 5 days after to confirm the effectiveness of the infection. Mice were then treated with cumanin or benznidazole, the drug currently used to treat infected humans, for 5 consecutive days. Parasitemia and survival were periodically recorded. **Figure 7A** shows how cumanin was able to dramatically reduce the number of circulating parasites in the acute phase of *T. cruzi* infection compared with the PBS-treated control. When we analyzed the area under the parasitemia curve (AUC) we observed an important decrease in the number of circulating parasites in cumanin treated mice comparing with controls (AUC: 382 and 800, respectively). This protection was more important at the peak of parasitemia, on day 22 postinfection, when control mice showed 8 times more parasites than cumanin-treated ones (8.2 and 1.2×10^7^ parasites/ml, respectively, p<0.01). As shown in **Figure 7A**, parasitemia values were slightly higher than those obtained with benznidazole but no significant differences were found between the two compounds.

The ability of cumanin to control infection was also reflected in the significant survival of the treated mice, respect to the control (p = 0.0086). **Figure 7B** shows the important reduction in mice mortality observed in cumanin-treated mice, where 100% of PBS-treated mice died between days 22 and 26 postinfection, while 66% of cumanin-treated mice survived the *T. cruzi* infection by the end of the experiment on day 100 (not shown). Again, cumanin-treated mice did not show any significant differences with respect to those treated with the approved drug for this parasitosis.

### *In vivo* toxicity assay

Hepatic toxicity of the STL was evaluated through the determination of a panel of hepatic-linked enzyme markers. Serum levels of AST, LDH and AST were measured at 7 days post treatment. **Table 2** shows that cumanin treated mice exhibited similar levels of the analyzed enzymes to those of PBS-treated control mice, suggesting that cumanin is not hepatotoxic *in vivo* at the doses used.

**Table 2:** Serum levels of hepatic enzymes in mice at 7 days after treatment with cumanin and PBS.

	LDH (IU/L)	AST (IU/L)	ALT (IU/L)
**Cumanin**	38.7±21.5	4.8±1.7	11.1±1.8
**PBS**	45.7±4.4	9.7±3.2	10.3±3.5

Results are expressed as mean ± S.E.M. (n = 5).

## Discussion

In a previous investigation we have reported the isolation of STLs from Argentinean *Ambrosia* species, some of which have shown significant trypanocidal and leishmanicidal activities [20]–[22]. These promising results prompted us to continue with the search for other antiprotozoal compounds from *Ambrosia scabra* and its related species, *A. elatior*.

The organic extract of *A. elatior* showed significant activity against *T. cruzi*, being able to inhibit 94% of epimastigote growth at a concentration of 100 µg/ml. When this extract was chromatographed on a Silica gel column, 9 final fractions (F_1AE_–F_9AE_) were obtained. Fractions F_5AE_, F_6AE_ and F_7AE_ displayed the highest trypanocidal activity against the non-infective form of *T. cruzi* and afforded compound A. The structural elucidation of this substance was based on the analysis of its spectral data and was identified as cumanin, which has been previously reported in *Ambrosia artemisiifolia* [28] and *Ambrosia cumanensis* [29].

We recently found that the F_5AS_ fraction of the organic extract of *A. scabra* was very active against *T. cruzi*. From F_5AS_ fraction we isolated the STL psilostachyin C, whose anti-epimastigote and anti-trypomastigote activity had been previously reported [22]. In this manuscript we report that further isolation steps from *A. scabra* fraction F_5AS_ yielded other three compounds (B, C, D). Compounds B and C were identified as the STLs, psilostachyin and cordilin, respectively, and compound D as daucosterol. The identification of all these compounds was performed by analysis of their spectral data. Psilostachyin had been previously isolated from *A. tenuifolia* by our group [20]; however, this is the first report of its presence in *A. scabra*, and the first time the isolation of cordilin and daucosterol from *A. scabra* has been reported. Psilostachyin and cordilin are diasteroisomers that only differ in the spatial configuration of the hydroxyl and methyl groups on C-5 (**Figure 1**).

*T. cruzi* is genetically highly diverse [30]. While RA, belonging to TcII-DTUs, is a highly virulent pantropic/reticulotropic strain, K98 (TcI) is a low virulence myotropic strain [31]. In order to analyze the relevance of cumanin, cordilin and daucosterol in different *T. cruzi* populations, K98 and RA strains were evaluated for their trypanocidal activity. Even though the epimastigote stage of the parasite is non-infective, it is easy to cultivate and therefore, it is useful for a preliminary screening test [32]. Cumanin exerted significant *in vitro* trypanocidal activity against this parasite form, showing an IC_50_ value 12 µM and 4 µM for RA and K98 strains. Cordilin was less active with an IC_50_ 26 µM and 44 µM, for the two strain analyzed. Daucosterol displayed even lower trypanocidal activity. However, in the search for new potential trypanocidal candidates, it is necessary to analyze the activity of the compounds on the infective forms of the parasite. Consequently, cumanin, cordilin and daucosterol were tested against trypomastigotes and all the isolated compounds were evaluated against intracellular amastigotes, including psilostachyin that had not been previously evaluated against this parasite form [20]. Cordilin and cumanin showed low activity on trypomastigotes (IC_50_ = 89 µM and 180 µM, respectively. The two STLs psilostachyin and cumanin inhibited the growth of the intracellular forms of *T. cruzi* with IC_50_s values of 21 µM and 8 µM, respectively. Cordilin and daucosterol did not display any activity against amastigotes.

The fact that cumanin is active against amastigotes is of particular interest, since the DNDi organization prioritizes the development of drugs that are useful during the indeterminate and chronic phases of the infection where parasites remain intracellular [33]. The ability of compounds to inhibit the intracellular growth of *T. cruzi* amastigotes is a more rigorous and relevant test of anti-*T. cruzi* activity, as it is applied to a stage which is the predominant and replicative form in mammals cells. The impairment of amastigotes replication upon cumanin treatment could lead to a reduction in tripomastigotes release from the cells and the subsequent low parasitemia observed in mice.

Nowadays, the association of compounds could be an interesting strategy for the control of Chagas disease. Thus, psilostachyin and cumanin were tested together on epimastigotes to assess their possible interaction. An additive effect was observed between these two compounds.

Considering the incidence of the *Leishmania* spp. in South America and the existence of patients co-infected with these parasites and *T. cruzi* [34]–[36], the effect of the four compounds on *L. braziliensis* and *L. amazonensis* promastigotes was also evaluated. Cumanin, psilostachyin and cordilin revealed significant leishmanicidal activiy against both *L. amazonenzis* and *L. brazilensis*, while daucosterol leishmanicidal activity was lower.

Trypanocidal activity of STL is highly influenced by stereochemical or structural differences [37]. Schmidt et al. [38] demonstrated that the STL helenalin was more active against *T. cruzi* and *T. b. rhodesiense* than its diasteroisomer mexicanin, which differs from the former only in the spatial orientation of an OH group. In the case of the two epimeric STLs isolated from *A. scabra*, cordilin and psilostachyin, the behavior of the two compounds against non-infective and infective forms of *T. cruzi* was very different. We have previously reported that psilostachyin is very active against epimastigotes and trypomastigotes showing IC_50_ values of 4 µM and 3 µM, respectively [20]. Those values compared with the results herein reported for cordilin with values of 26 µM (epimastigotes) and 89 µM (trypomastigotes) signal that psilostachyin is more active than cordilin. In the present study, we have also demonstrated that psilostachyin is more active than its 5-epimer on the intracellular form of the parasite. Thus, these results support the fact that stereochemistry plays an important role in the biological activity.

The administration of cumanin to *T. cruzi*-infected mice (1 mg/kg/day) produced a significant reduction in parasitemia levels, even lower than that produced by benznidazole, when compared with PBS-treated control mice. An 8-fold reduction in parasitemia levels was observed on day 22 postinfection compared with control. Moreover, cumanin was able to reduce the number of parasites during the whole course of the infection. More importantly, 66% of the mice survived the deadly challenge with trypomastigotes while only 20% of benznidazole-treated mice survived, compared with 100% mortality in the PBS-treated mice group. It should be noted that the dosis of 1 mg/kg/day used was irrespective of cumanin and benznidazole IC50 values.

Interestingly, cumanin is more active on intracellular amastigotes than on free trypomastigotes, which is not the case for psylostachyin, since it is very active in both stages (**Figure 5** and reference [20]). This phenomenon could be attributed to the fact that cumanin differently affects both parasite stages somehow interacting in different metabolic pathways that deserve to be further investigated. An alternative hypothesis suggesting that cumanin affects both parasite stages similarly as well as the cells infected by amastigotes, consequently killing the cell host and parasites, cannot be sustained since cumanin showed to be nontoxic for non-infected cells at the concentration used (**Table 2**).

The determination of *in vitro* and *in vivo* toxicity is a very important point in a drug discovery process [17]. Cumanin and cordilin displayed low cytotoxicity on Vero cell line at concentrations of up to 100 µg/ml (data not shown). Moreover, cumanin-treated mice (non-infected) exhibited similar serum levels of hepatic-linked enzyme markers than those in the PBS-treated control mice (**Table 2**). Thus, by using *in vitro* and *in vivo* toxicity assays, we demonstrated the non-toxic effect of cumanin at the doses used. These results are in agreement with those reported by Lastra et al. [39], who found that cumanin produces low cytotoxicity on peritoneal murine macrophages.

As it was mentioned in the introduction section, different types of terpenoid compounds have shown to display antiprotozoal activity [18], [40], and among them, several STLs having an α,β-unsaturated-γ-lactone moiety have been reported to show trypanocidal and leishmanicidal activities [18], [41], [42]. Thus, the STLs psilostachyin and cumanin can be considered interesting lead molecules for the development of drugs for Chagas' disease. Variability in the outcome and morbidity of *T. cruzi* infection might be associated, at least in part, with the complex population structure of the parasite. Taking this into account, the fact that cumanin shows high activity at different stages and strains of *T. cruzi* highlights the importance of searching new drugs against Chagas disease. In addition, the leishmanicidal activity shown by these compounds in a preliminary assay could be an interesting fact to consider, since the endemic areas of Chagas disease and leishmaniasis usually overlap in Latin America and co-infected patients have been reported [34]–[36].

Despite the advances in the biology of protozoan parasites like *T. cruzi* and *Leishmania* sp., the discovery and development of safer drugs to treat American trypanosomiasis and/or leishmaniasis still constitute a challenge. The results presented in this work demonstrate that terpenoids, particularly STLs, are an interesting group of natural compounds that could become good candidates for antiprotozoal chemotherapy. Further studies involving the evaluation of different targets will be useful to understand the mechanisms of action of the isolated STLs.
